# Explainable deep learning approaches and clinical insights for cancer biomarker identification

**DOI:** 10.3389/fonc.2026.1810793

**Published:** 2026-04-23

**Authors:** Kshitij Srivastava, Ruby Srivastava

**Affiliations:** 1 Department of Psychiatry, Ganesh Shankar Vidyarthi Memorial (GSVM) Medical College, Kanpur, Uttar Pradesh, India; 2 Department of Chemistry, Indian Institute of Technology Bombay, Mumbai, India

**Keywords:** biomarkers, black box, deep learning, explainable AI, immunotherapies

## Abstract

Biomarkers play a pivotal role in contemporary cancer immunotherapy by guiding diagnosis, patient stratification, therapeutic decision-making, and longitudinal assessment of treatment responses. Despite the transformative impact of immune checkpoint inhibitors, adoptive cell therapies, and neoantigen-based vaccines, durable clinical benefit is achieved in only a subset of patients, highlighting the critical need for accurate predictive and prognostic biomarkers. Technological advances are rapidly expanding the biomarker repertoire through high-resolution approaches such as single-cell and spatial omics, circulating tumor DNA analysis, immune-related gene expression signatures, and microbiome profiling. These platforms enable deeper characterization of immune dynamics, resistance mechanisms, and therapeutic responsiveness. Recent advances in artificial intelligence, machine learning, and deep learning have fundamentally reshaped immunotherapy biomarker discovery by enabling the integration of complex, high-dimensional multiomics, radiomic, and clinical datasets into unified predictive frameworks. Deep learning models have demonstrated superior performance in predicting immune checkpoint inhibitor responses, immune-related adverse events, and mechanisms of therapeutic resistance across multiple cancer types. The incorporation of explainable AI approaches further enhances clinical interpretability by linking algorithmic predictions to biologically validated immune processes. Future progress will depend on multimodal biomarker integration, analytical standardization, and rigorous prospective validation, alongside addressing regulatory, economic, and implementation challenges to advance precision cancer immunotherapy.

## 1 Introduction

Cancer represents one of the most pressing global health challenges, accounting for approximately 10 million deaths annually worldwide (1). The rising incidence of cancer is driven by multiple factors, including population growth, increased life expectancy, and exposure to risk factors such as tobacco use, unhealthy dietary habits, environmental pollution, and sedentary lifestyles (2). The low and middle-income countries bear a disproportionate share of global cancer burden, contributing to nearly 70% of cancer-related mortality. Commonly affected organs include the lungs, breast, colon, brain, and stomach. Although lifestyle modifications can substantially reduce cancer incidence, effective screening and early detection programs remain inaccessible or inadequately implemented in many regions (3). Consequently, the escalating global cancer burden underscores the urgent need for safe, cost-effective, accessible, and efficient strategies for cancer prevention, early diagnosis, and treatment (4). The integration of artificial intelligence (AI) into oncology has emerged as a transformative approach to cancer diagnosis, prognosis, and therapeutic decision-making. It aligns with the growing emphasis on precision medicine, which considers not only disease characteristics but also individual genetic profiles, environmental exposures, lifestyle factors, and clinical history when selecting optimal treatments (5). One of the most impactful applications of AI in precision oncology is biomarker discovery, a cornerstone for personalized cancer care.

A biomarker is defined as “a measurable characteristic that reflects normal biological processes, pathogenic processes, or responses to therapeutic interventions” (6, 7). The FDA-NIH Biomarkers, EndpointS, and other Tools (BEST) classification categorizes biomarkers into seven major groups: susceptibility or risk biomarkers, which indicate disease predisposition; diagnostic biomarkers, which detect disease presence or subtype; monitoring biomarkers, which assess disease status over time; prognostic biomarkers, which predict disease outcome; predictive biomarkers, which forecast treatment response; pharmacodynamic or response biomarkers, which demonstrate biological response to therapy; and safety biomarkers, which indicate the risk or occurrence of treatment-related toxicity (6, 8). These biomarker classes provide valuable insights across different stages of disease progression and clinical management. Additionally, diagnostics serve as essential medical tools for guiding the safe and effective use of specific therapies by identifying responders and monitoring potential adverse effects (9). The growing complexity of biomarker discovery is largely driven by the increasing diversity of biomedical data types. Traditional biomarkers typically rely on single measurable parameters, such as glycated hemoglobin (HbA1c) or cholesterol levels. Glycated hemoglobin (HbA1c) was first noticed more than 40 years ago as an unusual type of hemoglobin in people with diabetes. A few small studies showed that HbA1c levels are closely related to blood glucose levels, so now HbA1c is used as a test to diagnose diabetes, but only when proper quality checks are followed and the test is standardized according to international guidelines (10).

The advances in high-throughput technologies have generated high-dimensional datasets that require advanced computational approaches for analysis. These datasets encompass multiple biological and clinical data modalities. Omics-based data include genomics, epigenomics, transcriptomics, proteomics, glycomics, metabolomics, and microbiomics. The integration of multiple omics layers has gained significant attention, particularly in single-cell analysis, where multiple molecular profiles can be obtained from individual cells (11–15). Traditionally, biomarker discovery has relied on hypothesis-driven approaches, which are increasingly inadequate given the complexity of cancer biology and the vast volume of data generated by modern high-throughput ‘omics’ technologies (16). Conventional indicators including PD-L1 expression, tumor mutational burden (TMB), microsatellite instability (MSI) or mismatch repair (MMR) deficiency, tumor-infiltrating lymphocytes (TILs), and tumor neoantigen burden (TNB) remain central to clinical practice but inadequately reflect the dynamic and heterogeneous nature of tumor–immune interactions (16).

The rapid advancement of artificial intelligence (AI), particularly machine learning (ML) and deep learning (DL) techniques is revolutionizing biomarker discovery by enabling the extraction of meaningful patterns from large-scale, multidimensional datasets. These approaches facilitate the identification of complex and non-intuitive biomarker signatures across multiomics data, thereby enhancing cancer screening, diagnosis, and prognosis (17). AI-driven biomarkers play a pivotal role in informing patient-specific treatment responses, especially in cancer immunotherapy, aiding therapy selection, predicting disease progression, and monitoring treatment outcomes. The incorporation of AI into biomarker research is expected to significantly improve diagnostic accuracy, particularly by overcoming longstanding limitations in biomarker sensitivity and specificity.

**Figure 1:**
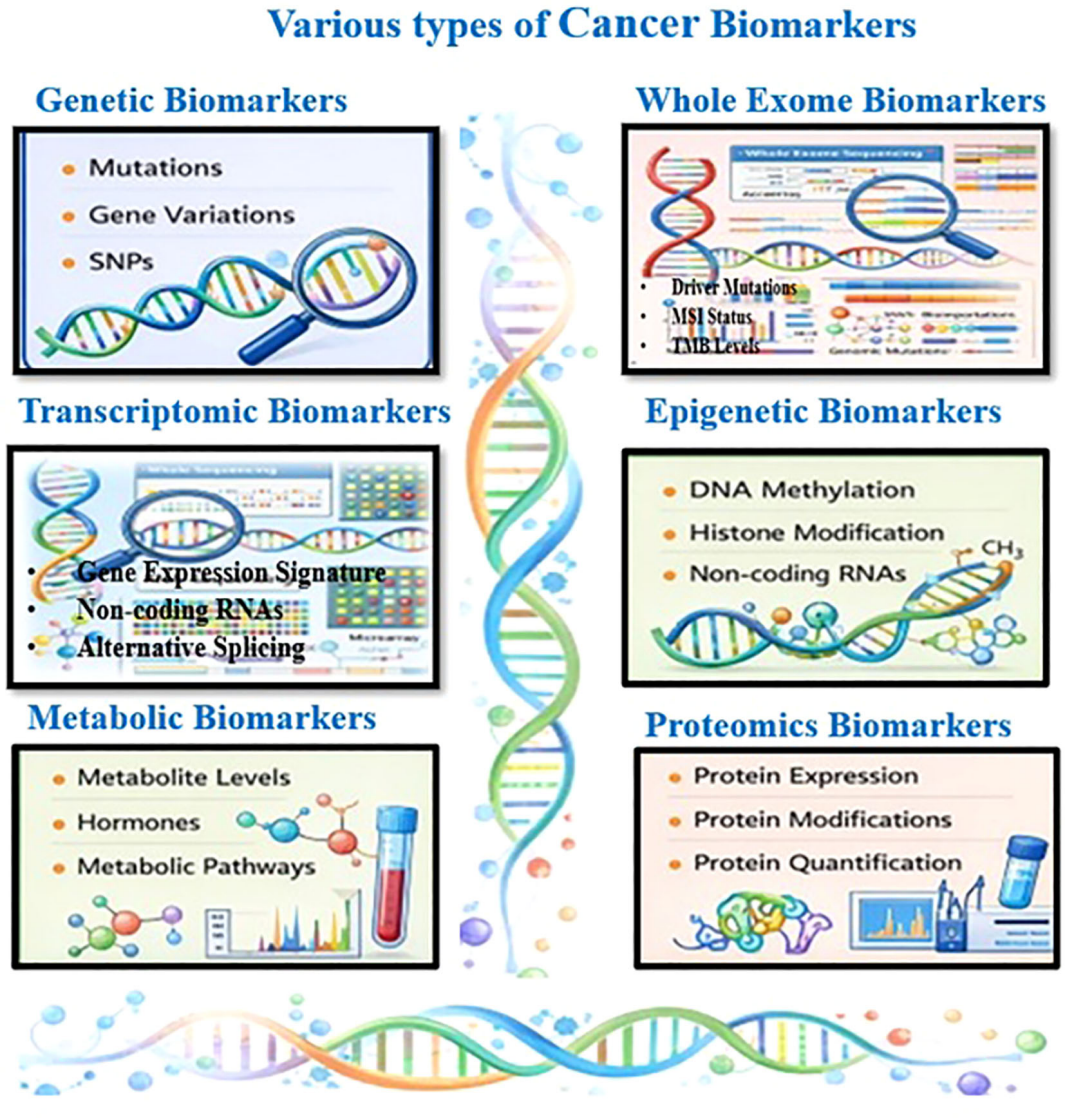
A comprehensive overview of different types of cancer biomarkers.

Sensitivity refers to a biomarker’s ability to correctly identify affected individuals (true positives), whereas specificity denotes its capacity to correctly exclude unaffected individuals (true negatives). Both metrics are essential for evaluating diagnostic performance. Conventional cancer screening tools, such as mammography for breast cancer and prostate-specific antigen (PSA) testing for prostate cancer, often suffer from false-positive (FP) or false-negative (FN) results, leading to overtreatment or missed diagnoses (17). In contrast, AI-based models can identify highly specific biomarker signatures associated with distinct cancer subtypes, thereby improving diagnostic reliability. By analyzing complex histopathological images, AI systems have demonstrated enhanced diagnostic accuracy and consistency. Numerous studies have shown that ML and DL models effectively distinguish cancerous tissues from healthy samples, contributing to more precise and individualized treatment strategies (18, 19). AI-driven analyses of multi-omics data, radiomics, pathomics, and clinical records have proven effective in predicting cancer onset and progression, as demonstrated by Ozaki et al. (20). ML and DL algorithms have shown superior performance in classifying cancer types and stages, particularly in breast, lung, brain, and skin cancers (21). DL models trained on extensive histopathological image datasets frequently achieve diagnostic accuracy comparable to or exceeding that of expert pathologists. When integrated with molecular biomarker data, these image-based analyses provide a comprehensive diagnostic framework that significantly enhances early detection. In lung cancer, the AI-assisted diagnostics combining radiological and genomic data have yielded promising results, enabling earlier intervention and improved patient outcomes (22). A clear and simple overview of the various types of cancer biomarkers is given in **Figure 1**.

Beyond diagnosis, AI-derived biomarkers possess substantial prognostic and predictive value. These biomarkers enable oncologists to anticipate patient outcomes and tailor treatment strategies accordingly. This capability is particularly critical in cancer immunotherapy, where patient responses vary considerably. AI models can identify biomarker signatures predictive of responsiveness to immune checkpoint inhibitors, facilitating personalized treatment selection and improved therapeutic efficacy (23). Additionally, AI-based prognostic biomarkers offer dynamic insights into tumor evolution by detecting subtle temporal changes in patient data, such as circulating tumor DNA or RNA levels, allowing early identification of disease recurrence or therapeutic resistance before clinical symptoms emerge (24). This real-time predictive capability empowers clinicians to proactively adjust treatment regimens, potentially improving survival rates and quality of life. Over the past 15 years, treatment strategies for solid tumors have significantly evolved with the immune checkpoint inhibitors and targeted therapies. Despite the crucial role of next-generation sequencing in identifying actionable genomic alterations, its routine clinical use is limited by high costs and technical complexity, restricting comprehensive genomic profiling for many patients (25, 26). The growing complexity of treatment selection has led to the development of multidisciplinary tumor boards to guide patient stratification and therapy decisions, though access remains limited in smaller healthcare settings, causing treatment delays. Moreover, many patients either do not respond to immunotherapy and targeted therapies or eventually develop resistance. While biomarkers such as MSI, PD-L1 expression, and tumor mutational burden provide some predictive value, their accuracy remains inconsistent, and targeted therapies still lack reliable markers to identify non-responders. Although molecular profiling has enabled the identification of emerging DNA and RNA-based biomarkers, many candidates lack sufficient clinical validation, highlighting the need for more advanced and integrative biomarker discovery approaches capable of capturing complex biological mechanisms underlying treatment response (27–29). Further clinical insights for biomarker identification bridge advanced computational modeling with actionable bedside decisions, ensuring that biomarker-driven predictions translate into clear, high-impact clinical indications that directly inform patient selection and therapeutic management.

## 2 AI frameworks for cancer biomarkers

The identification of tumor antigens and the development of cancer vaccines have emerged as promising strategies in modern cancer immunotherapy. Somatic mutations that are selectively expressed in cancer cells can give rise to neoantigens following intracellular processing and presentation. Because neoantigens are recognized by CD4^+^ and CD8^+^ T cells without undergoing central immune tolerance, they represent highly specific and immunogenic targets for T cell–based immunotherapies (30). Owing to their patient-specific nature, neoantigen-based therapies inherently support personalized treatment paradigms. Neoantigens arise from tumor-specific genetic alterations, and their identification has been greatly accelerated by the application of AI to high-throughput sequencing data (31–33). Weber et al. developed a ML–based computational pipeline, EasyFuse, which processes transcriptomic data to detect cancer-associated gene fusions that may serve as highly immunogenic neoantigen sources (34) Beyond single-modality approaches, multimodal integration of genomics, transcriptomics, and proteomics data has led to the development of advanced ML frameworks such as NeoDisc. This system demonstrated superior performance over conventional pipelines in accurately prioritizing immunogenic neoantigens (35). Effective neoantigen presentation requires binding to major histocompatibility complex (MHC) molecules, making peptide–MHC binding prediction a critical step in tumor vaccine design (36). To address this challenge, transfer learning models such as MHCRoBERTa have been developed using large-scale, label-agnostic protein sequence datasets (37). Additionally, integrated ML frameworks like Anthem combine mass spectrometry data with computational modeling to predict human leukocyte antigen class I (HLA-I) binding with high accuracy (38). DL–based platforms, including ImmuneApp, have also been proposed for HLA-I binding prediction (39). For HLA class II (HLA-II) binding, Racle et al. implemented a ML framework capable of achieving robust predictive performance (40). By simulating immune system interactions and optimizing antigen selection, AI plays a pivotal role in the rational design of personalized cancer vaccines. These computational approaches help prioritize the most promising vaccine candidates and improve the efficiency of vaccine development pipelines. For example, AI-assisted mRNA and peptide vaccine designs developed by Powderly et al. and Xu et al., respectively, have progressed to phase I clinical trials across multiple cancer types (41, 42). Moreover, adjuvants are critical components of cancer vaccines, enhancing immune activation and therapeutic efficacy (43). AI contributes to adjuvant discovery through virtual screening, molecular property prediction, rational design, and drug repurposing strategies (44). In a study, three potent CXCL12 inhibitors were identified with potential adjuvant activity using ligand-based virtual screening approaches (45).

Long non-coding RNAs (lncRNAs) are key regulators of transcriptional control, epigenetic modifications, and post-transcriptional processes, and they play a significant role in tumor immune regulation and the tumor microenvironment (TME) (46). AI-based methods have facilitated deeper insights into lncRNA-mediated mechanisms and their therapeutic potential in cancer immunotherapy. Numerous studies have shown that lncRNAs interact with RNA-binding proteins, and DL approaches have been instrumental in elucidating these interactions and identifying novel RNA-based therapeutic targets (47).

Since tumor-infiltrating lymphocytes (TILs) has a critical role in immunotherapy response, immune cell–associated lncRNA signatures in low-grade glioma and tumor-infiltrating B lymphocytes is developed (48). By integrating ML algorithms with clinical data, these studies demonstrated that immune-related lncRNA signatures can effectively identify patients most likely to benefit from immunotherapy. Similarly, Liu et al. applied integrative ML strategies to validate the clinical relevance of lncRNAs in colorectal cancer (49). Their consensus immune-related lncRNA signature successfully predicted patient outcomes, revealing differential therapeutic benefits between bevacizumab and fluorouracil-based chemotherapy. A subsequent consensus ML derived lncRNA signature reported by Liu et al. further supported these findings and demonstrated additional utility in predicting tumor recurrence (50).

## 3 Methodology

AI was formally introduced as a scientific discipline at the 1956 Dartmouth Conference, marking the beginning of systematic research into intelligent machines (51). DL, a specialized branch of ML, has existed conceptually since the 1980s; however, it gained widespread prominence after 2006 due to substantial advances in computational power and the availability of large-scale datasets. By leveraging multi-layer artificial neural networks, DL models learn hierarchical representations of data, achieving high predictive performance across diverse domains such as computer vision, natural language processing (NLP), and intelligent engineering systems (52, 53). DL models differ fundamentally from traditional computational approaches in several key aspects, including feature extraction, data and computational requirements, interpretability, and problem-solving paradigms. DL architectures automatically learn relevant features directly from raw data, eliminating the need for manual feature selection (54). However, this advantage comes at the cost of increased data and computational demands. DL models typically require large, well-annotated datasets and extensive computational resources, including high-performance graphics processing units (GPUs) and substantial memory and storage capacity, to achieve superior performance (54, 55). Another major distinction lies in model interpretability. DL models are frequently described as “black boxes” due to their complex architectures and large numbers of interconnected parameters, which make manual interpretation and parameter adjustment challenging (56, 57). DL has shifted the paradigm toward data-driven learning, allowing models to infer representations directly from large collections of sample data rather than relying on predefined physical assumptions (58, 59). In recent years, these advantages have positioned DL as a powerful and increasingly indispensable tool in oncology. Integrating multi-omics data has therefore become essential for gaining deeper insights into tumor biology, improving diagnostic accuracy, and enabling personalized treatment strategies. However, the rapid growth of high-dimensional omics datasets presents significant challenges related to data heterogeneity, noise, and integration complexity. DL offers an effective solution by handling high-dimensional, heterogeneous, and unstructured data and by uncovering nonlinear and multimodal relationships that are difficult to detect using conventional analytical methods (60, 61). Consequently, DL has demonstrated strong potential across various stages of cancer management, including early detection and screening, diagnosis, molecular subtype classification, prognosis estimation, survival prediction, and evaluation of treatment response (62).

At the core of DL based data integration are neural network (NN) architectures composed of several fundamental components. These include an input layer, which receives raw data such as images, text, or numerical features; multiple hidden layers, which perform nonlinear transformations using convolutional, recurrent, or fully connected units to extract hierarchical features; and an output layer, which generates task-specific predictions. Pooling layers are often incorporated to reduce feature dimensionality, merge semantically similar features, lower computational costs, and mitigate overfitting. Model parameters, including weights and biases, are optimized through backpropagation during training. Activation functions introduce nonlinearity, enabling networks to model complex relationships, while loss functions such as mean squared error or cross-entropy quantify discrepancies between predicted and actual outcomes. Optimizers iteratively update network parameters to minimize loss functions, and regularization techniques introduce constraints to reduce overfitting and improve model generalizability. The DL based integration of multi-omics data typically follows a structured workflow consisting of six key stages: data pre-processing, feature selection or dimensionality reduction, data integration, model construction, data analysis, and result validation. Data pre-processing is a critical initial step and involves cleaning and standardizing datasets to address issues such as missing values, noise, and redundancy. Common pre-processing methods include imputation of missing values, removal of outliers, and normalization techniques such as z-score or min–max scaling, all of which enhance data quality and integration reliability (63). Given the high dimensionality of multi-omics datasets, feature selection or dimensionality reduction is often required to reduce computational complexity and minimize redundancy. Techniques such as principal component analysis (PCA) and autoencoders (AEs) are widely employed for this purpose. PCA performs linear transformations to project data into a lower-dimensional space while preserving variance, whereas AEs learn compact, nonlinear representations of data through NN architectures. These approaches not only improve computational efficiency but also reduce the risk of overfitting. Data integration involves combining information from multiple omics layers into a cohesive analytical framework and can be implemented using early, intermediate, or late integration strategies (64). Early integration concatenates raw omics features prior to dimensionality reduction, intermediate integration merges feature after omics-specific pre-processing, and late integration combines results obtained from independent analyses of each omics layer. The choice of integration strategy depends on data characteristics and analytical objectives, with effective integration maximizing information sharing across datasets to enhance model robustness and predictive accuracy.

Model construction represents the core analytical step and requires selecting an appropriate DL architecture based on data type and task objectives, such as classification, regression, or clustering. Common architectures include convolutional neural networks (CNNs), recurrent neural networks (RNNs), and AEs, each suited to specific data structures and analytical goals (65). CNNs are particularly effective for image-based data, capturing spatial patterns, while RNNs are well suited for sequential or time-series data, modeling temporal dependencies. During the analysis phase, trained DL models are applied to integrated datasets to perform tasks such as disease classification, biomarker prediction, and patient subgroup identification. Finally, model performance must be rigorously evaluated using appropriate validation metrics. Common evaluation measures include accuracy and F1 score for classification tasks, mean squared error (MSE) for regression analyses, and the silhouette coefficient for clustering applications (66). The selection of evaluation metrics should align with the specific analytical objectives and dataset characteristics. These metrics provide quantitative measures of model effectiveness and inform further optimization and refinement (67). As computational power continues to grow, DL architectures have become increasingly complex, further exacerbating interpretability challenges. This complexity arises from the intricate interactions among multiple nonlinear layers, large numbers of trainable parameters, and high-dimensional input data, making it difficult to trace how specific inputs contribute to final predictions. These limitations hinder clinical trust and pose barriers to the safe deployment of DL systems in healthcare applications.

To further improve interpretability and transparency, the concept of explainable artificial intelligence (XAI) has emerged, which made the AI systems more understandable and trustworthy for clinicians and patients (68). XAI seeks to mitigate the black-box nature of complex DL models by providing interpretable representations of how decisions are made, thereby increasing confidence in AI-driven clinical decision support systems (69). XAI methods can be categorized into two main classes: model-based and *post hoc* approaches (70). Model-based interpretability techniques are inherently transparent by design and provide direct insight into learned relationships between input variables and outcomes, as exemplified by linear and logistic regression models. In contrast, *post hoc* explainability methods are applied to complex, non-interpretable models and aim to extract meaningful explanations after training, often through feature attribution, example-based reasoning, or visualization techniques such as saliency mapping (70).

**Figure 2:**
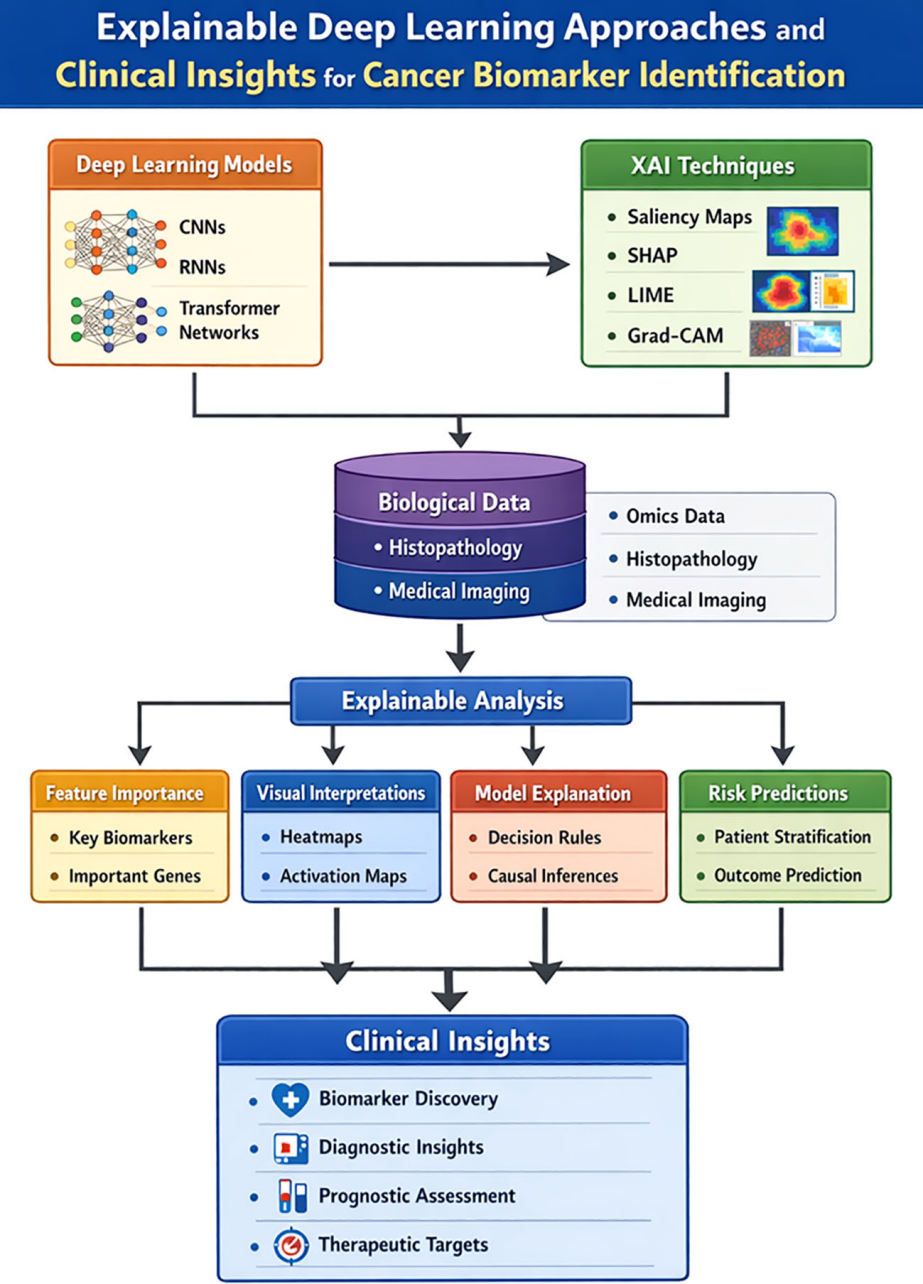
Overview of XAI and clinical insights for biomarker cancer identification.

A range of interpretability techniques including Local Interpretable Model-Agnostic Explanations (LIME) and SHapley Additive exPlanations (SHAP) provide insights into model predictions by estimating the contribution of individual input features. Despite their widespread adoption, these approaches have inherent limitations. LIME generates locally faithful explanations that may not reflect the global behavior of the model and is known to suffer from instability, where repeated explanations using the same settings can produce divergent results. Similarly, SHAP values, although grounded in game theory, may be computationally intensive and can be sensitive to model assumptions, particularly in high-dimensional settings. These methods further improve interpretability by quantifying the influence of specific clinical, molecular, or imaging features on prediction outcomes, increasing transparency and reliability. Visualization-based approaches, such as Gradient-weighted Class Activation Mapping (Grad-CAM), enable intuitive interpretation of DL models by highlighting image regions that most strongly influence predictions. In cancer imaging, these heatmaps help clinicians understand which tumor regions contribute to malignancy detection or subtype classification, thereby enhancing diagnostic confidence (71). The workflow of XAI approaches and clinical insights for identification of cancer biomarkers is given in **Figure 2**.

XAI has demonstrated substantial utility across multiple cancer research applications, including tumor detection and classification, biomarker identification, treatment response prediction, and patient survival analysis. In radiological and histopathological image analysis, XAI helps clinicians understand the rationale behind model classifications, supporting more accurate and explainable diagnoses. In personalized treatment planning, XAI highlights the patient-specific features that most strongly influence therapeutic decisions, facilitating tailored interventions and advancing precision medicine. XAI enhances the transparency of AI-assisted cancer care and supports more informed clinical decision-making (72). Beyond clinical utility, explaining model decision-making processes provides valuable biological insights and supports scientific discovery by revealing previously unrecognized associations within complex datasets. The interpretability is also essential for establishing the trustworthiness of AI models, which is critical for their successful deployment among end-users, including clinicians and patients.

The comparison of these approaches can be summarized in two main trade-offs: performance vs. interpretability and model complexity vs. clinical usability. These approaches should not rely on a single method but instead combine their strengths. In this next-generation framework, DL is used to detect complex biological patterns, multi-omics integration helps us understand the disease at a system-wide level, and XAI makes the results clear and trustworthy for clinicians. By bringing these approaches together, the AI systems can be built that are not only accurate but also understandable, reliable, and practical for real clinical use in precision oncology. See **Table 1**.

**Table 1:** List of various XAI approaches with its characteristics, strengths, limitations, best use cases and critical insights.

Approach	Characteristics	Strengths	Limitations	Best use cases	Critical insight
Machine Learning (ML)	Relies on handcrafted features; simpler models (e.g., SVM, RF)	Interpretable; low computational cost; works with small datasets	Limited ability to capture nonlinear and high-dimensional relationships	Structured clinical data; small datasets	Good baseline models but insufficient for complex multi-omics data
Deep Learning (DL)	Multi-layer neural networks; automatic feature extraction	High predictive accuracy; captures complex patterns; handles unstructured data (images, genomics)	Requires large datasets; computationally expensive; black-box nature	Imaging, genomics, survival prediction	Powerful but lacks transparency; needs XAI for clinical trust
Early Integration (Multi-omics)	Combines raw features from all omics layers before modeling	Maximizes data utilization; captures cross-modal interactions	High dimensionality; noise accumulation; risk of overfitting	Large, well-curated datasets	Information-rich but sensitive to noise and scale
Intermediate Integration	Extracts features per modality, then integrates	Balanced approach; reduces noise; improves robustness	Requires careful feature engineering or representation learning	Heterogeneous multi-omics datasets	Most practical trade-off between performance and stability
Late Integration	Combines outputs of separate models for each omics layer	Modular; interpretable; flexible	Misses cross-modal feature interactions	Independent analysis pipelines	Easier to implement but less biologically integrative
Model-based XAI	Inherently interpretable models (e.g., linear regression)	Transparent; easy to understand	Limited modeling capacity; oversimplifies biology	Clinical decision support with simple features	High trust but low complexity handling
*Post hoc* XAI (LIME, SHAP)	Explains predictions after model training	Model-agnostic; flexible; widely applicable	Computationally expensive; unstable (LIME); assumption-sensitive (SHAP)	Feature importance analysis	Improves interpretability but may not fully reflect model behavior
Visualization-based XAI (Grad-CAM)	Heatmaps highlighting important regions in images	Intuitive; useful for imaging tasks	Qualitative; lacks quantitative rigor	Radiology, histopathology	Enhances clinician trust but needs validation
DL + XAI Integration	Combines predictive power with interpretability tools	Balances accuracy and explainability; clinically relevant	Still evolving; lacks standardization	Precision oncology, biomarker discovery	Most promising direction for clinical translation

## 4 Discussion

AI-driven integrative models are reshaping how we understand tumor biology by moving beyond isolated data types toward a unified, systems-level view of cancer. Instead of relying on single-modality biomarkers, these approaches combine imaging, molecular, and clinical data to capture both the visible characteristics of tumors and their underlying biological drivers. Since cancer is inherently heterogeneous; no single data source can fully describe its complexity. Multimodal AI models address this gap by linking radiological and histopathological features (e.g., from CT, MRI, PET, and whole-slide images) with genomic and transcriptomic information, thereby improving diagnostic accuracy and prognostic prediction compared with traditional approaches (28, 73). A key insight emerging from this work is that imaging is no longer just descriptive but increasingly predictive of molecular states. Radiomics and DL methods extract high-dimensional features such as texture, shape, and spatial heterogeneity that reflect tumor microenvironmental complexity. These features enable AI systems to perform tasks like tumor segmentation, staging, and treatment monitoring with high precision. DL models can infer hidden biological information, including driver mutations and molecular subtypes, directly from images (67). This establishes a critical conceptual bridge between phenotype and genotype: tumors that look similar under imaging may behave differently at the molecular level, and AI helps uncover these hidden distinctions. The integration of imaging and molecular biomarkers therefore enables earlier detection of subtle lesions, more accurate prediction of disease progression and survival, and better personalization of therapy (73–76).

Real-world applications reinforce this conceptual shift. In lung cancer, AI-based radiomics models have improved early detection by identifying subtle imaging patterns missed by radiologists (77). In breast cancer, DL-based pathology tools have surpassed human-level accuracy in detecting small or ambiguous malignant regions, reducing diagnostic variability (78). Similarly, platforms integrating genomic and clinical data have improved treatment selection by identifying predictive biomarkers for targeted and immunotherapies (79). In non-small cell lung cancer (NSCLC), this integrative paradigm is particularly evident (80). Studies combining histopathology, PET/CT imaging, and molecular profiling have achieved high accuracy in distinguishing subtypes such as Lung adenocarcinoma (LUAD) and lung squamous cell carcinoma (LUSC) using both classical ML and DL approaches (80–83). These examples indicates that AI is not merely assisting clinicians but actively augmenting decision-making by integrating diverse data streams into clinically actionable insights.

DL models such as InceptionV3 have demonstrated the ability to predict gene mutations directly from histopathological images, achieving high Area Under the Receiver Operating Characteristic Curve (AUROC) values and revealing that visual tumor features encode genomic information (81). Parallel molecular studies using gene expression profiling and feature selection methods have also achieved high classification accuracy (84–88). As the imaging and molecular approaches converge toward the same biological signals, it suggests that AI can unify these domains into a single predictive framework. In few studies, the XAI-based feature selection has shown superior performance over traditional methods while identifying biologically meaningful biomarkers (89–92). Validation strategies such as cross-validation, external cohort testing, and survival analysis further strengthen the clinical relevance of these findings (93–95).

The ability of DL models is to infer molecular biomarkers directly from routine histopathology slides. Since hematoxylin and eosin (H&E) slides are widely available, this approach offers a cost-effective alternative to expensive molecular testing. DL models have successfully predicted mutations, microsatellite instability (MSI), tumor mutational burden (TMB), and protein expression levels across multiple cancer types (96–98). These findings suggest that much of the molecular information required for precision oncology may already be embedded in standard diagnostic images, waiting to be extracted computationally. However, challenges such as tumor heterogeneity, dataset variability, and limited generalizability across populations remain significant barriers (99, 100). AI has also enabled new biological insights rather than just improved prediction. For example, DL-based analysis of MSI and TMB has revealed associations between tumor heterogeneity and clinical outcomes that are difficult to detect using conventional assays (101–105). Similarly, spatial transcriptomics combined with DL has uncovered complex gene expression patterns linked to prognosis (106, 107). AI is not demonstrated only as a diagnostic tool but also a discovery platform capable of revealing previously unrecognized relationships within tumor biology. The multimodal AI architectures such as graph-based and attention-based models (e.g., MOGONET, DeepKEGG, DeepSSC) are enabling integration across genomics, transcriptomics, and epigenomics (108–113). Coupled with advances in foundation models for pathology and large language models for clinical data interpretation, these systems are moving toward fully automated, end-to-end precision oncology pipelines (114–116). The segmentation models like nnU-Net and SAM are improving tumor delineation and response assessment, further enhancing clinical workflows (117–123).

The unique contribution of AI is not just improved accuracy, but the ability to connect different biological layers imaging, molecular, and clinical into a coherent framework. However, achieving true clinical translation will require overcoming challenges in interpretability, standardization, and validation across diverse patient populations. Further progress in AI-driven oncology depends on moving from retrospective model development to prospective, clinically embedded validation. Integrating AI biomarkers into clinical trials, electronic health records (EHRs), and real-world evidence frameworks will be essential for demonstrating utility and reproducibility. Regulatory approvals of AI-based diagnostic tools and the adoption of digital pathology workflows already indicate growing clinical acceptance. At the same time, combining XAI with multimodal learning will enable transparent decision-making and facilitate clinician trust. The convergence of AI, large-scale biomedical data, and clinical implementation strategies is expected to transform precision oncology from a reactive to a predictive and preventive discipline, where treatment decisions are guided by continuously learned, data-driven insights (117–120).

A study developed and validated an interpretable ML model leveraging circulating tumor DNA (ctDNA) to predict progression-free survival (PFS) in patients with non-small cell lung cancer (NSCLC) undergoing immunotherapy, addressing limitations of conventional biomarkers such as PD-L1 expression and tumor mutational burden. Using pretreatment ctDNA data from 441 patients across the OAK trial, POPLAR trial, and a multicenter retrospective cohort, a 5-fold cross-validated LASSO-Cox model identified 25 key genomic features, which were integrated into an XGBoost framework. The model demonstrated strong predictive performance (AUCs: 0.82, 0.79, and 0.77 across training, validation, and test cohorts, respectively) and effectively stratified patients by survival outcomes. Key biomarkers included TP53 mutations associated with poorer prognosis and BRCA2 mutations linked to improved outcomes, while SHAP analysis highlighted NOTCH1 as a potential novel biomarker with immunomodulatory relevance. Overall, the model showed significant clinical utility, outperforming conventional treatment strategies (124). In a neoadjuvant immunotherapy (NIT), AI-driven models are increasingly used to support personalized treatment strategies. Current approaches can be broadly classified into two paradigms: indirect and direct prediction. The indirect paradigm focuses on estimating established surrogate biomarkers, such as PD-L1 expression and tumor mutational burden, to infer therapeutic response, whereas the direct paradigm utilizes AI to analyze large-scale molecular and clinical data to identify novel, data-driven biomarkers that directly predict clinical outcomes. Together, these complementary strategies highlight the transformative role of AI in enhancing biomarker discovery and improving response prediction in NIT. Similarly, explainable ML frameworks applied to large real-world datasets of over 38,000 patients have shown that AI-derived composite biomarkers outperform single-marker strategies like PD-L1 in predicting immunotherapy response, highlighting the clinical limitations of existing FDA-approved biomarkers when used in isolation (125).

Beyond lung cancer, XAI-driven analyses in ovarian cancer have successfully identified and validated clinically used biomarkers such as CA125, HE4, and CEA while simultaneously uncovering their nonlinear risk thresholds, thereby enhancing interpretability and clinical applicability in diagnostic workflows (126). Keyl et al. present an XAI-based analysis of real-world data from over 15,000 patients spanning 38 solid cancer types, demonstrating the potential of AI-driven biomarkers for clinical decision support by integrating multimodal data including clinical records, imaging-derived body composition, and tumor mutational profiles. The study identified 114 key prognostic markers that accounted for the majority of the model’s predictive capacity and revealed over 1,300 significant interactions between these variables. The XAI framework further enabled patient-level interpretation of prognostic contributions, enhancing transparency and clinical relevance. The findings were validated in an independent cohort of more than 3,000 lung cancer patients from a nationwide US electronic health record database, underscoring the robustness and generalizability of the approach (127).

Accurate prognostication is critical for guiding clinical management in localized cutaneous melanoma (CM), the deadliest form of skin cancer, yet conventional staging systems such as the American Joint Committee on Cancer (AJCC) staging system rely on limited tumor characteristics and often overlook key clinicopathological factors and the tumor microenvironment (TME). In this context, the multimodal AI model MelanoMAP integrates TME-derived digital biomarkers with clinicopathological features from more than 3,500 histology slides to enhance prognostic accuracy. The model demonstrated strong performance, achieving a C-index of 0.82 significantly outperforming traditional staging (0.66) and showed consistent superiority across multiple international cohorts. Explainability analysis using SHAP further revealed that TME-derived digital biomarkers, along with established factors such as age, mitotic count, and Breslow depth, are critical determinants of metastatic risk. MelanoMAP highlights the potential of AI-driven digital biomarkers to improve personalized prognostication and support clinical decision-making in melanoma (128).

Ghosh et al. (129) developed an explainable AI framework using Natural Language Processing (NLP) to improve patient matching for early-phase oncology studies such as Phase 1 clinical trials, addressing key challenges in recruitment efficiency. The prototype system matches patient records with trial protocols based on four criteria—cancer type, performance status, genetic mutations, and measurable disease and generates both a composite matching score and interpretable evidence. Evaluation against expert-annotated ground truth using a dataset of 12 synthetic patient records and 6 trial protocols demonstrated promising performance, with a precision of 73.68%, recall of 56%, accuracy of 77.78%, and specificity of 89.36%. Analysis of misclassifications revealed that abbreviation ambiguity and contextual misunderstanding were primary error sources, while the system correctly identified no-match scenarios in all false positive cases. Across 21 ML studies in pancreatic cancer, only a small subset incorporated XAI, primarily through methods such as SHAP and SurvSHAP. These approaches enabled the identification of key biomarkers, comorbidities, and survival predictors while improving clinician trust in model outputs. XAI techniques were broadly categorized based on their implementation stage (ante-hoc vs. *post-hoc*), compatibility (model-agnostic vs. model-specific), and explanatory scope (local vs. global). Despite their promise, several barriers to clinical adoption remain, including methodological instability, limited external validation, poor integration into clinical workflows, and the absence of standardized evaluation frameworks (130).

Breast cancer is a highly heterogeneous malignancy with significant mortality, where early diagnosis and precise subtype classification are critical. Leveraging multi-omic data, particularly DNA methylation, this study introduces a two-stage explainable AI framework, XAI-MethylMarker, to identify clinically relevant biomarkers. In the first stage, a DL model (MethylNet) combines an autoencoder for dimensionality reduction with a feed-forward neural network to classify breast cancer subtypes. In the second stage, an explainable biomarker discovery algorithm (MethylBDA) applies multiple XAI techniques to extract a concise set of 52 biomarkers. The model achieved a classification accuracy of 0.8145 ± 0.07 (95% confidence interval) using 5-fold cross-validation. Gene set analysis further validated the clinical relevance of these biomarkers, identifying 14 druggable genes, nine prognostic genes, and several significantly enriched pathways associated with distinct breast cancer subtypes, underscoring the potential of XAI-driven approaches in precision oncology (131). Another study focuses on the identification and application of novel genetic biomarkers for hepatocellular carcinoma using XAI, addressing the limitations of conventional biomarkers such as Alpha-fetoprotein, which often lack sufficient sensitivity and specificity. The authors developed a multi-model XAI framework integrated with probabilistic causal inference to analyze clinical and gene expression data, enabling the discovery and validation of prognostically significant biomarkers. The approach demonstrated strong predictive performance and clinical relevance through interpretable metrics, identifying key genes including TOP3B, SSBP3, and COX7A2L as consistently influential across multiple models. These biomarkers showed potential to enhance prognostic accuracy beyond AFP, with particular relevance highlighted for the Hispanic population, supporting the importance of demographic-specific precision oncology research (132).

## 5 Challenges in AI-driven biomarker discovery

Despite the rapid progress in AI-driven biomarker discovery, several challenges remain, particularly in malignancies such as ovarian and pancreatic cancers, where limited data availability and heterogeneity restrict model development and validation. To address these limitations, innovative computational strategies are being developed. AI research are proposed to enhance data interoperability while preserving patient privacy and introduced advanced Bayesian models capable of detecting cancer at early stages using longitudinal clinical data (133). Nevertheless, concerns regarding AI transparency, interpretability, and clinical reliability persist and must be addressed to ensure safe and effective implementation in healthcare settings. Data-related challenges remain a primary limitation. Robust AI models require large, high-quality, and well-annotated datasets; however, the availability of diverse and representative clinical data across geographic and demographic populations is limited (134). Dataset quality directly influences model reliability, while insufficient diversity can result in biomarkers that lack generalizability and may exacerbate existing healthcare disparities (135). Ethical concerns further complicate data use, particularly regarding patient privacy and data security, as AI development often involves large-scale aggregation of sensitive medical information. Statistical uncertainties arising from data variability, small sample sizes, and model assumptions may also compromise diagnostic performance. Addressing these issues requires robust data collection pipelines, cross-validation strategies, uncertainty quantification, XAI, and continuous model updating to improve reliability and clinical trust. Interpretability and transparency pose additional challenges. Many AI and DL models function as “black boxes,” making it difficult to interpret their decision-making processes. This opacity undermines clinician confidence, particularly in oncology, where treatment decisions are often life-critical (136). The lack of explainability complicates clinical adoption, emphasizing the need for interpretable and transparent AI systems that clinicians can trust and validate. AI-identified biomarkers must undergo rigorous validation to ensure reliability, reproducibility, and applicability across diverse patient populations. However, many AI models lack extensive experimental or prospective clinical validation, raising concerns about generalizability (137). Integrating AI tools into established clinical workflows also presents logistical challenges, while navigating complex regulatory approval pathways often delays implementation (138, 139). Failure to resolve these concerns risks reinforcing socio-economic and geographic healthcare disparities rather than alleviating them. Major technical challenges include imbalanced datasets, heterogeneous molecular profiles, and limited availability of labeled data. These issues impair biomarker identification and model performance. Potential mitigation strategies include dataset integration, advanced ML techniques, and the use of DL architectures capable of handling high-dimensional medical data. DL models, particularly NN, excel at processing complex data types such as medical images and electronic health records, enabling early disease detection and prediction of treatment response.

Regulatory hurdles, the need for large-scale clinical validation studies, and integration into routine clinical workflows remain significant obstacles. Effective implementation also requires training healthcare professionals, establishing clear clinical guidelines for DL use, and continuous model monitoring. Importantly, AI systems should support not replace clinical expertise, preserving physician accountability in medical decision-making. DL models in medical diagnostics face additional vulnerabilities, including susceptibility to noisy data, annotation errors, and adversarial attacks that may compromise patient safety. Data noise from imaging variability, human error, and inconsistent diagnostic criteria introduces bias and limits generalizability. To address these issues, improved data quality management, robust training methodologies, adversarial defense strategies, ensemble learning, and continuous clinical validation are essential. Recent studies have emphasized solutions to data privacy, bias, and fairness in AI-driven cancer diagnostics. Tasci et al. (140) identified class imbalance as a major source of algorithmic bias in oncological datasets and proposed mitigation strategies. McGraw (141) highlighted the importance of diverse datasets and expert involvement throughout AI development and deployment to reduce bias in radiology applications. Clinical informatics approaches including real-world data analysis, natural language processing, and radiomics have been employed to identify and mitigate cancer disparities across demographic groups; however, careful assessment of algorithmic bias remains essential to avoid reinforcing existing inequalities (142). Malin and Goodman et al. (143) further discussed balancing data accessibility and privacy using consent frameworks, privacy risk assessment, cryptographic querying, and game-theoretic methods for secure data sharing. These studies underscore the necessity of addressing bias and privacy to ensure equitable and effective cancer care.

The practical implementation of XAI in real-world oncology settings presents both significant opportunities and substantial challenges. While XAI has the potential to improve clinician trust and support decision-making by providing transparent insights into model predictions, its integration into routine clinical workflows remains complex. One major challenge is the lack of standardized metrics to evaluate the quality and clinical usefulness of explanations, making it difficult to compare methods and ensure reliability across different healthcare settings (144). Additionally, the inherently heterogeneous nature of oncology data spanning imaging, genomics, and clinical records complicates the development of XAI systems that are both accurate and interpretable, often forcing a trade-off between model performance and explainability (145). Practical deployment is further hindered by difficulties in integrating XAI tools into time-sensitive clinical workflows, where systems must be intuitive, fast, and minimally disruptive to existing practices (146). From a clinical perspective, explanations must also be tailored to different users, as what is meaningful to data scientists may not align with clinicians’ reasoning or decision-making processes (147). Moreover, ethical and regulatory concerns including accountability, patient safety, and data privacy pose additional barriers, particularly when incorrect or misleading explanations could impact clinical outcomes (148). Finally, limited real-world validation, lack of usability studies, and insufficient evidence on how XAI influences clinical decisions further restrict widespread adoption (149). Addressing these challenges will require standardized evaluation frameworks, user-centered design, multidisciplinary collaboration, and rigorous clinical validation to ensure that XAI systems are not only technically robust but also clinically meaningful and trustworthy.

## 6 Addressing challenges

The challenges associated with DL and XAI based models for cancer biomarker discovery can be addressed through a combination of robust methodological design, biological validation, and transparent model interpretation. Data heterogeneity and limited sample sizes can be mitigated by integrating multi-omics datasets, employing transfer learning, and using standardized pre-processing pipelines to improve model generalizability. Model overfitting and poor reproducibility may be reduced through rigorous cross-validation, external cohort validation, and detailed reporting of training strategies. The black-box nature of DL models can be addressed by incorporating XAI techniques such as SHAP, LIME, and attention mechanisms to enhance interpretability and align computational outputs with known cancer and immune biology. Additionally, bias arising from imbalanced datasets should be systematically evaluated using subgroup analyses and fairness-aware learning approaches. Importantly, embedding clinician-in-the-loop frameworks and validating AI-derived biomarkers in experimental and clinical settings are essential to ensure reliability, translational relevance, and clinical adoption.

## 7 Conclusion and future outlook

Currently, approximately 2,000 biomarkers have been identified, yet only about 6% are routinely used in clinical practice. A major limitation is poor reproducibility, often resulting from inadequate preanalytical and analytical rigor, limited biomarker validation, and flawed study designs. Insufficient evaluation of false positives and false negatives particularly when comparing patient cohorts with healthy controls further restricts clinical applicability. A comprehensive understanding of biomarker validation methodologies is therefore essential for selecting clinically relevant and reliable markers. The integration of ML and DL into biomarker research is accelerating discovery, improving early detection, and enabling personalized therapeutic strategies. These approaches enhance predictive accuracy beyond traditional methods, deepen understanding of cancer biology, and improve patient risk stratification. AI has become a cornerstone of modern biomarker discovery, giving rise to AI-powered biomarkers and opening new avenues for scientific innovation. Emerging concepts such as “Virtual Labs” coordinated systems of AI agents functioning as virtual scientists demonstrate the potential for automated, multi-modal biomedical discovery. AI-driven automation may enable large-scale, multi-modal biomarker discovery, integrating imaging, omics, clinical data, and real-time sensor inputs to support precision medicine. However, widespread adoption will require interdisciplinary collaboration among biologists, clinicians, computational scientists, and AI engineers. Crucially, AI-powered biomarkers must be interpretable and explainable to gain clinical acceptance (133). Advances in wearable and sensor technologies will further support continuous, real-time health monitoring (150). To maximize societal benefit, these innovations must be democratized to reduce health inequities and ensure global accessibility. Future progress in AI-enabled medical diagnostics depends on targeted strategies to overcome existing limitations. These include enhanced data collection through augmentation, active learning, and improved annotation workflows; adversarial training to improve robustness; ensemble approaches to reduce diagnostic errors; and transparency-focused XAI methods to build clinician trust. Transfer learning and few-shot learning approaches can mitigate data scarcity, enabling model adaptation with limited labeled datasets. Furthermore, extensive validation across diverse populations is critical to ensure clinical effectiveness. Collaborative research and data-sharing initiatives are essential to expand access to high-quality medical datasets and improve global health outcomes.
